# The Roles of Host 5-Methylcytosine RNA Methyltransferases during Viral Infections

**DOI:** 10.3390/ijms21218176

**Published:** 2020-10-31

**Authors:** Maciej Wnuk, Piotr Slipek, Mateusz Dziedzic, Anna Lewinska

**Affiliations:** Department of Biotechnology, Institute of Biology and Biotechnology, University of Rzeszow, 35-310 Rzeszow, Poland; piotrslipek1@gmail.com (P.S.); mateusz.m.dziedzic@gmail.com (M.D.)

**Keywords:** 5-methylcytosine, NSUN proteins, DNMT2/TRDMT1, viruses, antiviral drugs

## Abstract

Eukaryotic 5-methylcytosine RNA methyltransferases catalyze the transfer of a methyl group to the fifth carbon of a cytosine base in RNA sequences to produce 5-methylcytosine (m^5^C). m^5^C RNA methyltransferases play a crucial role in the maintenance of functionality and stability of RNA. Viruses have developed a number of strategies to suppress host innate immunity and ensure efficient transcription and translation for the replication of new virions. One such viral strategy is to use host m^5^C RNA methyltransferases to modify viral RNA and thus to affect antiviral host responses. Here, we summarize the latest findings concerning the roles of m^5^C RNA methyltransferases, namely, NOL1/NOP2/SUN domain (NSUN) proteins and DNA methyltransferase 2/tRNA methyltransferase 1 (DNMT2/TRDMT1) during viral infections. Moreover, the use of m^5^C RNA methyltransferase inhibitors as an antiviral therapy is discussed.

## 1. Introduction

The development of molecular techniques has allowed for research intensification in the field of RNA modifications. Up to now, over a hundred of RNA modifications have been identified including the methylation of fifth carbon in cytosine catalyzed by a number of m^5^C RNA methyltransferases [1]. Since the first report demonstrating chromatography-based detection of methylated cytosines in poly(A)-messenger RNA [2], m^5^C was also detected in other types of RNA such as transfer RNA (tRNA), ribosomal RNA (rRNA), small nuclear RNA (snRNA), microRNA (miRNA), long noncoding RNA (lncRNA), transactivation response element (TAR), small vault RNA (vtRNA), and enhancer RNA (eRNA) of many organisms across all phylogenetic groups [3,4,5,6] Thanks to advances in RNA bisulfite sequencing, methylated RNA immunoprecipitation sequencing, and 5-azacytidine-mediated RNA immunoprecipitation or methylation-induced cross-linking immunoprecipitation, new qualitative and quantitative information about the target sites of RNA cytosine methyltransferases may be provided [7,8,9,10]. Molecular studies have also shown that m^5^C can regulate many aspects of RNA metabolism, namely, RNA export, ribosome assembly, translation, and RNA stability [11]. However, the impact of RNA cytosine methylation on transcriptional and translational efficiency during viral infection is still far from being understood. More information is available concerning the effects of N^6^-methyladenosine (m^6^A) and 2′O-methylated (Nm) ribonucleotides. For instance, m^6^A may stabilize the viral RNA and efficiency of viral protein translation [12,13]. Moreover, the 2′-O-methylation on the first nucleotide of the viral RNA may protect the viral RNA from the innate immune response pathway by camouflaging the viral RNA as a cellular mRNA [14]. Despite the role of m^6^A and 2′O-methylated (Nm) ribonucleotides in viral RNA metabolism is well characterized, the function of m^5^C during viral infection is less explored, and important issues remain to be resolved [15]. Therefore, here, we summarize the latest findings concerning the roles of m^5^C RNA methyltransferases during viral infections and suggest the use of m^5^C RNA methyltransferase inhibitors in antiviral therapies.

## 2. m^5^C RNA Methyltransferases and Their RNA Targets

It is widely accepted that in humans, m^5^C is incorporated into different RNA molecules by the action of seven members of the NOL1/NOP2/SUN domain (NSUN) family of proteins, namely, NSUN1, NSUN2, NSUN3, NSUN4, NSUN5, NSUN6, and NSUN7, and the DNA methyltransferase 2/tRNA methyltransferase 1 (DNMT2/TRDMT1) [16]. Moreover, selected NSUN proteins such as NSUN1, NSUN2, and NSUN5 are evolutionally conserved and, e.g., the yeast *Saccharomyces cerevisiae* orthologues are available, namely, Nop2, Trm4, and Rcm1, respectively [6]. m^5^C RNA methyltransferases rely on *S*-adenosylmethionine (SAM) as a donor of the methyl group, and NSUN proteins utilize two catalytic cysteines in the active site, whereas DNMT2 uses a single cysteine in the active site that is similar to the mechanism of action of other DNA methyltransferases [17,18]. Regardless of mechanism involved, a covalent intermediate is created between a protein cysteine and the cytosine in RNA to stimulate the electron-deficient pyrimidine heterocycle for the nucleophilic attack of fifth carbon on the methyl group of SAM that is located in two conserved motifs IV and VI in NSUN family members [17].

**Figure 1:**
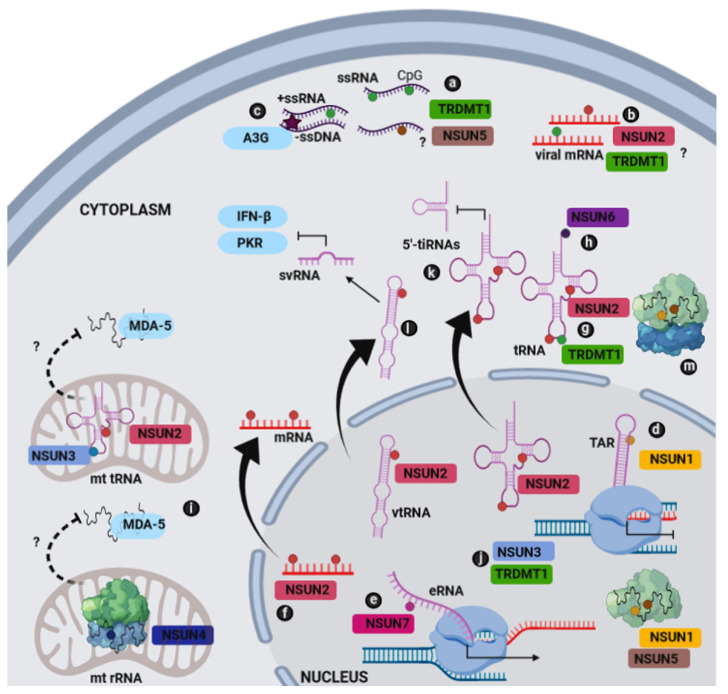
Overview of human m^5^C RNA methyltransferases and their RNA targets during viral infections. (**a**) The methylation of 5′C—phosphate—G3′ (CpG) islands in viral genomic RNA by DNMT2 (TRDMT1) and perhaps NSUN5 may promote viral heterogeneity. (**b**) The modification of viral transcriptome by NSUN2 and perhaps TRDMT1 may result in increased transcript stability and efficient translation. (**c**) The cytosine deamination by A3G may lead to dC-to-dU conversions in viral (–)ssDNA that may cause G-to-A hypermutations in progeny viral genomes. (**d**) Induction of viral latency by NSUN1-mediated m^5^C methylation of TAR. (**e**) NSUN7-mediated m^5^C methylation of eRNA, a transcriptional coactivator of PGC-1α (peroxisome proliferator-activated receptor gamma coactivator 1-alpha) and an activator of RNA polymerase II. (**f**) NSUN2-mediated methylation of viral mRNA promotes its splicing and transport into the cytoplasm (a black arrow). (**g**,**h**) NSUN2-, TRDMT1-, and NSUN6-mediated methylation of tRNA molecules protects them against stress-induced degradation and supports efficient translation. (**i**) Putative protective role of NSUN2-, NSUN3-, and NSUN4-mediated methylation of tRNA and rRNA in the mitochondria. These modification events prevent cytoplasmic translocation of tRNA and rRNA and binding of stress-based degraded form of tRNA and rRNA to a pattern-recognition receptor for RNA (MDA-5) that in turn counteracts the induction of a type I interferon response. (**j**) NSUN3 and TRDMT1 as putative regulators of RNA polymerase II-mediated gene transcription during cellular stress response. (**k**) NSUN2-mediated biogenesis of tiRNAs. (**l**) NSUN2-mediated methylation of vtRNA may promote its conversion to svRNA that, in turn, may inhibit the translation of PKR and IFN-β production. (**m**) NSUN1- and NSUN5-mediated methylation of rRNA supports ribosome biogenesis and efficient translation. rRNA, ribosomal RNA; tRNA, transfer RNA; mt tRNA, mitochondrial tRNA; eRNA, enhancer RNA; svRNA, specific vtRNA-derived small non-coding RNA; vtRNA, vault RNA; TAR, transactivation response element; tiRNA; tRNA-derived stress-induced RNA, mRNA, messenger RNA; ssRNA, single-stranded RNA; MDA-5, melanoma differentiation-associated protein 5; PKR, RNA-dependent protein kinase; IFN-β, interferon beta; ?-putative action, A3G, apolipoprotein B mRNA editing enzyme catalytic subunit 3G (cytidine deaminase); *- dC-to-dU conversion in viral negative-sense single-stranded DNA (**–ssDNA**).

Different subcellular localization and RNA targets of human m^5^C RNA methyltransferases have been documented (Figure 1) [6]. Nucleolar NSUN1 (NOP2/nucleolar antigen p120) methylates the single C5 position of cytosine (4447nt) in 28S rRNA [19]. Nuclear/nucleolar NSUN2 introduces m^5^C at two positions of intron-containing tRNA(Leu)(CAA) precursors (34 and 48nt) and at three positions of tRNA(Gly)(GCC) precursors (48, 49, and 50nt) [20,21] as well as in mitochondrial tRNAs [22]. NSUN2 may be also detected in the cytoplasm. NSUN2 also methylates cytosine to produce m^5^C in various RNAs such as mRNAs, lncRNAs, and small non-coding RNAs, such as vault RNAs (vtRNAs) [20,21,23,24]. Mitochondrial NSUN3 mediates methylation of cytosine to yield m^5^C at the nucleotide 34 of mt-tRNA(Met) [25,26]. Mitochondrial NSUN4 methylates mt-12S rRNA at the nucleotide 841 [27]. Nucleolar NSUN5 methylates the single C(5) position of cytosine in 28S rRNA (3782nt) [28,29,30]. Cytoplasmic and Golgi apparatus located NSUN6 methylates the C5 position of cytosine 72 in tRNA(Thr)(TGT) and tRNA(Cys)(GCA) [31,32,33]. Nuclear NSUN7 methylates enhancer RNAs (eRNAs) at different positions [34]. Cytoplasmic, and to a less extent nuclear, DNMT2 (TRDMT1) methylates the aspartic acid, glycine, and valine tRNA at the cytosine-38 residue in the anticodon loop [35,36].

Diverse phenotypic features have been reported in mice lacking selected NSun proteins. NSun1 deletion induced apoptotic cell death, affected lineage specification and decreased the pools of RNA during mouse embryonic development [37]. In NSun2^−/−^ mice, testis size is decreased and sperm differentiation is inhibited [38]. Moreover, Dnmt2^−/−^NSun2^−/−^ embryos were smaller and lighter compared to their wild-type littermates. Newborn double-knockout mice also appeared smaller at birth, failed to develop visible milk spots, showed a reduced thickness and organization of the cerebral cortex and an immature skeleton with incomplete ossification [39]. Dnmt2^−/−^NSun2^−/−^ mice were also characterized by changes in cellular lipid storage [39]. It has been documented that Nsun4 is essential for embryonic development in the mouse [27]. The growth of mutant embryos (Nsun4^−/−^) was significantly retarded [27]. Moreover, knockout of Nsun4 in the mouse heart resulted in mitochondrial dysfunction, namely, mitoribosome assembly and mitochondrial translation were diminished [27]. In contrast, adult Nsun5-KO mice displayed spatial cognitive deficits with impairment of oligodendrocyte precursor cells [40]. Nsun5 knockout in mice also resulted in a decrease in body weight and lean mass and limited protein synthesis and growth [30]. It has been suggested that NSun6 is dispensable for mouse embryonic development, and loss of NSUN6-associated methylation lowers mRNA stability in embryonic cells [41]. Mice with mutated Nsun7 gene showed male sterility as a consequence of affected sperm motility [42].

## 3. Host-Based Methylation of the Fifth Position of Cytosine Residues in Viral RNA

In 1975, the presence of 5-methylcytosine (m^5^C) in the Sindbis virus 26S RNA obtained from infected host cells was revealed and the putative role of m^5^C during viral infection was postulated [43,44]. Similar observations were noticed in the case of adenovirus 2 [45]. In the RNA of cells infected with adenovirus 2, N^6^-methyladenosine (m^6^A) and a minor fraction of m^5^C were detected. However, the authors were unable to identify the presence of m^5^C in the viral RNA particles.

Recent studies using ultrahigh-performance liquid chromatography (UHPLC), tandem mass spectrometry (UPLC-MS/MS), and photo-cross-linking-assisted m^5^C sequencing have revealed that genomic RNA of murine leukemia virus (MLV) contains higher levels of m^6^A, m^5^C, and 2′O-methylated (Nm) ribonucleotides than cellular mRNA [46]. Similarly, 10-fold higher levels of m^5^C and 2′O-methylations have been observed in human immunodeficiency virus 1 (HIV-1) RNA compared to these noticed in typical cellular mRNAs [47]. Courtney et al. determined that m^5^C accounted for 0.6–1.4% of cytosines in HIV-1 RNA (approximately 11 m^5^Cs per HIV-1 genomic RNA) [47].

It is worthwhile to mention that methylation occurs the most frequently in CpG regions of DNA and RNA viruses [48,49,50]. More recently, it has been shown that CpG may be also over-represented in dsRNA viruses at the location across codon boundaries compared to negative-sense single-stranded RNA (−ssRNA) and positive-sense single-stranded RNA (+ssRNA) viruses [51]. It is postulated that the changes in retroviral CpG RNA may be a result of cytidine deaminase activity. However, the mechanism that may contribute to the elimination of CpG in riboviruses is still unknown. Perhaps it is not based on cytidine deaminase activity as riboviral RNA does not form the DNA intermediate that is essential for cytidine deaminase activity [51,52].

The analysis of RNA modification using direct RNA sequencing also revealed m^5^C signals in viral RNA of *Coronaviridae* family [53,54]. More recently, a comprehensive study on severe acute respiratory syndrome coronaviruses (SARS-CoV) using direct RNA sequencing documented that 42 positions with predicted m^5^C modification occur at consistent positions between subgenome-length mRNAs [55]. Thus, one may ask a question if the occurrence of cytosine methylation at fifth position may be a source of genetic heterogeneity in early steps of coronavirus infection. This may rely on the discontinuous extension for synthesis of subgenome-length negative strands [56], namely, a nested set of 5′- and 3′-coterminal subgenomic (sg) mRNAs is produced and this set is characterized by a common 5′ leader sequence, which is identical to the 5′-end of the viral genome [57,58]. However, the role of 5-methylcytosine in viral genomic RNA, especially in *Coronaviridae* family, is not well established and requires further comprehensive studies.

It is known that experimental increase in the pools of CpG dinucleotides in CpG-deficient viral genomes may lead to significant decrease in viral replication and virulence [59,60,61]. Thus, it may suggest that m^5^C RNA methyltransferases may play an important role during the recognition of viral CpG and the inhibition of replication process of selected types of viruses. For example, fruit fly m^5^C RNA methyltransferases Dnmt2 has been reported to directly interact with *Drosophila* C virus (DCV) RNA being a part of antiviral defense strategy as an evolutionarily conserved innate immune response [62,63] (Figure 1).

It has been shown that NSUN5 can also bind to the core protein of HCV, a positive-strand RNA virus [64] (Figure 1). On the other hand, it has been reported that the methyltransferase NSUN2 may serve as the primary writer for m^5^C on HIV-1 genomic RNA [47]. Inactivation of NSUN2 resulted in limited addition of m^5^C to HIV-1 transcripts and inhibited viral replication [47]. Thus, we suggest that this modification may be important also for genomic RNA stabilization, RNA transport to host cellular compartments, replication regulation, protection against degradation, and promotion of viral genetic heterogeneity that is based on cytidine deaminase activity.

To the best of our knowledge, little is known about the effects of m^5^C RNA methyltransferases on the activity of cytidine deaminase-based antiviral defense system. The evolutionarily conserved APOBEC family of proteins (apolipoprotein B mRNA editing enzymes) are deaminase enzymes (cytidine-to-uridine editing enzymes) that allow for editing of RNA/ssDNA sequences and may promote diversity is mRNA editing [65]. Apolipoprotein B editing complex 3 (APOBEC3) consists of one gene in rodents and up to seven genes in primates, namely, *APOBEC3A*, *APOBEC3B*, *APOBEC3C*, *APOBECDE*, *APOBEC3F*, *APOBEC3G,* and *APOBEC3H* [65]. APOBEC3 subfamily has important role during viral infections as it can inhibit a number of viruses, e.g., HIV-1, human T-lymphotropic virus (HTLV), hepatitis C virus (HCV), hepatitis B virus (HBV), human papillomavirus (HPV), herpes simplex virus 1 (HSV-1) and Epstein-Barr virus (EBV) by editing-dependent and independent mechanisms [65]. In particular, APOBEC3G (A3G) promotes cytidine-to-uridine hypermutations during reverse transcription and deaminates C residues in CC motifs and other members of APOBEC3 group provide modifications in CT motifs [66,67]. APOBEC3 may promote beneficial mutations of HIV type-1 that may result in adaptation and evolution in natural infection [68]. APOBEC3A can be considered as a potent deamination factor of both C and m^5^C, while APOBEC3G is much weaker in its ability to deaminate m^5^C [69]. More recently, the N2-C271A NSUN2 mutant was considered to study the proteins, which are packaged into HIV-1 virions [47]. This mutagenic event is based on the substitution of second conserved cysteine to alanine that may result in spontaneous cross-links to target cytosines. Interestingly, APOBEC3G and NSUN2 were shown to be packaged into HIV-1 virions in the N2-C271A NSUN2 mutant [47]. This may suggest putative interactions between NSUN2 and APOBEC3G. However, the consequences of such interactions need to be determined in the future (Figure 1). Perhaps host m^5^C RNA methyltransferases may protect some viruses against mutagenic activity of cytidine deaminase that may limit cytidine deaminase-mediated lethality.

## 4. The Modulation of Host Cellular Metabolism via Virus Hijacking of RNA Processing

The transcriptome of host cells infected with several RNA viruses, e.g., Zika virus (ZIKV), dengue virus (DENV), HCV, poliovirus, and HIV-1, has been reported to be post-transcriptionally modified [70]. This may suggest that post-transcriptional modifications such as 5-methylcytosine may be considered as a new layer of regulation by which RNA viruses subvert the host and evade cellular surveillance systems [70].

Viral infections are also accompanied by cellular stress responses in the host cells that may affect proper transcription and translation of viral mRNA and proteins, respectively [71]. Thus, m^5^C RNA methyltransferases can be exploited to sustain viral transcriptional and translational activity by the incorporation of 5-methylcytosine into viral mRNA and/or host tRNA and rRNA [20]. Indeed, there are numerous examples of the involvement of host m^5^C RNA methyltransferases in RNA modification-based regulation of transcription and translation upon viral infections.

It has been postulated that NSUN1 (NOP2), a member of ribosomal large subunit assembly complex, may play the role in the regulation of the cell cycle and in the nucleolar activity associated with cell proliferation [72]. Kong et al. have recently shown that NOP2 restricts HIV-1 replication, suppresses HIV-1 transcription, and promotes viral latency via m^5^C methylation of TAR RNA [73] (Figure 1).

Depletion of NSUN7 decreased the levels of m^5^C in eRNA. Methylated eRNA is considered to be a transcriptional coactivator associated with peroxisome proliferator-activated receptor-gamma co-activator 1 alpha (PGC-1α) that modulates cellular metabolic responses. eRNA methylation stabilizes eRNA-bound protein complex and enhances RNA polymerase II activity [34]. This may suggest the involvement of NSUN7 during metabolic reprogramming of infected cells as PGC-1α is robustly induced upon HCV infection [74] (Figure 1).

NSUN2 is involved in various processes such as cellular differentiation by controlling protein synthesis, promoting tRNA stability and preventing mRNA decay [21]. It has been shown that HIV-1 mRNA in infected cells is highly modified by the addition of m^5^C by the host NSUN2 methyltransferase. Inactivation of NSUN2 resulted in the loss of m^5^C addition and inhibition of HIV-1 mRNA translation as well as reduced ribosome binding to viral mRNAs and the dysregulation of alternative splicing of viral RNAs [47] (Figure 1). It was also shown that downregulation of the m^5^C writer NSUN2 inhibits MLV replication [46].

It was also reported that human DNMT2 participates in RNA processing during cellular stress [75]. DNMT2 interacts with proteins involved in RNA processing and DNMT2 is translocated from the nucleus into the cytoplasm during cellular stress [75]. DNMT2 also promoted survival of HIV-1 RNA in infected host cells by RNA cytosine methylation activity [76]. HIV-1 induces DNMT2 translocation from the nucleus to the stress granules, and then, DNMT2 methylates HIV-1 RNA [76]. These suggest that HIV-1 ensures its own survival in the host cells via hijacking of the RNA processing machinery and stress granule promotion.

In contrast to DNMT2- and NSUN2-mediated tRNA modifications, the role of NSUN6 during viral infection remains elusive due to the lack of comprehensive research data. However, selected transcriptomic analyses showed that the changes in NSUN6 expression occur upon infection with the SARS-CoV [77] (Figure 1).

Thus far, there is no information regarding the direct associations between NSUN3 or NSUN4 and viral infection. However, it is evident that at least one role of these mitochondrial m^5^C rRNA methyltransferases is to maintain mitochondrial activity during infection-associated intense energy consumption [71] (Figure 1).

Interestingly, mRNA expression microarray datasets from the Gene Expression Omnibus (GEO) database showed that expression levels of both 5-methylcytosine rRNA methyltransferase genes in cells infected with Ebola virus (EBOV) (GSE17509), influenza A virus (IAV) subtype H5N1 (A/H5N1) (GSE43302), SARS-BatS RBD (GSE47960), and HCV JFH-1 strain (GSE20948) were altered suggesting that NSUN3 and NSUN4 proteins are active members of the cellular viral response pathway.

Here, we hypothesize that mitochondrial NSUNs are essential for the maintenance of mitochondrial RNA stability and loss of methylation may lead to the transfer of nonfunctional mtRNAs into the cytoplasm. mtRNAs can be recognized by MDA5 and bind to MDA5, a well-known pattern-recognition receptor for RNA that induces a type I interferon response [78]. This hypothesis is supported by a recent work of Dhir et al. (2018) showing that mitochondrial RNA could be a source of self-nucleic acids and an initiator of an interferon response via the cytoplasmic receptor MDA5 as a consequence of RNA metabolism [79] (Figure 1).

Moreover, NSUN3 may have distinct roles beyond the methylation of mt-tRNA(Met) I [25,26,80]. Cheng et al. have found that NSUN3 can form a complex with heterogeneous nuclear ribonucleoprotein K (hnRNPK), DNMT2, and positive transcription elongation factor b (P-TEFb) at elongating RNA polymerase II sites [80]. Thus, it is possible that DNMT2 and NSUN3 can be also considered as new regulatory proteins in transcriptional process in the nucleus during cancer progression or chronic cellular stress [80] (Figure 1).

## 5. Attenuation of Host Antiviral Response by Virus-Mediated Activation of NSUN2

NSUN2, a multifunctional methyltransferase, is also required for biogenesis of tRNA-derived non-coding fragments (tRFs) [20] (Figure 1). tRFs inhibit protein synthesis via several mechanisms including direct inhibition of the ribosome and displacement of RNA-binding proteins [81,82,83]. Interestingly, some viruses can use tRFs to modulate host immune responses [84]. Deng et al. showed that respiratory syncytial virus (RSV) induces activation of ribonuclease angiogenin (ANG) and cleaves mature cytoplasmic tRNAGlu (CTC) in its anticodon loop to produce two halves: 5-half (tRF5-GluCTC or 5-tiRNAGlu) and 3-half (3-tiRNAGlu) that suppress the expression of anti-RSV protein APOER2 [84].

In addition, C(5)-methylation of mRNAs regulates mRNA export (Figure 1). Methylated transcripts are specifically recognized by THOC4/ALYREF that mediates mRNA nucleocytoplasmic shuttling [85]. THOC4/ALYREF are essential for the export of Kaposi’s sarcoma-associated herpesvirus (KSHV) intronless mRNAs and infectious virus production via recruitment of the TREX complex [86].

NSUN2 controls processing of vault ncRNAs into small regulatory RNAs with microRNA functions such as the three vtRNAs [87]. vtRNA contains a cytosine methylation site, and the methylation of vtRNA1.1 promoted its processing into smaller fragments (svRNAs). The lack of NSUN2 protein leads to the loss of cytosine-5 methylation in vault RNAs causing aberrant processing into Argonaute-associated small RNA fragments [87]. vtRNAs can be also used by viruses to silence host antiviral responses [88]. The double-stranded (ds) RNA-dependent protein kinase (PKR), a member of host innate immune responses, mediates the activation of signal transduction pathways leading to interferon beta (IFN-β) gene induction during viral infection or RNA transfection [89] (Figure 1). It was shown that vtRNAs promoted viral replication in A549 cells and mouse lungs after infection with IAV through repressing PKR activation and the subsequent antiviral interferon response [88].

During SARS-CoV infection, small noncoding RNAs were also detected. The analysis of deep sequenced RNA isolated from the lungs of infected mice showed three 18–22 nt small viral RNAs (svRNAs). The three svRNAs were derived from the nsp3 (svRNA-nsp3.1 and -nsp3.2) and N (svRNA-N) genomic regions of SARS-CoV [90]. These observations suggest that the biogenesis of small viral RNA could be also a target of host NSUN2, but additional studies are required to confirm such assumptions.

## 6. Inhibitors of m^5^C RNA Methyltransferases as Antiviral Drugs

Taking into account the involvement of m^5^C RNA methyltransferases in the antiviral response, one can ask whether the inhibitors of these enzymes can be considered as antiviral drugs. Nucleotide and nucleoside analogs are examples of broad-spectrum antiviral drugs that inhibit transcription and/or replication of different RNA and DNA viruses [91]. Pharmacologic inhibition of DNA methylation has been successfully used as an anticancer therapy using the cytosine analogs 5-azacytidine (azacytidine) and the closely related compound 2-deoxy-5-azacytidine (decitabine) [92].

Interestingly, DNA demethylation responses of azacytidine and decitabine drugs are not restricted to the inhibition of DNA methylation [93]. Schaefer et al., using RNA bisulfite sequencing, showed that azacytidine, but not decitabine, inhibited cytosine-38 methylation of tRNA(Asp), a major substrate of DNMT2 [93]. They found that azacytidine caused a substantially stronger metabolic effect than decitabine in all cancer cell lines tested that is consistent with the effect of this drug on RNA metabolism [93]. In this context, it is interesting that 5-azacytidine has been shown to inhibit HIV-1 infectivity [94]. Molecular examination of the mechanism by which 5-azacytidine inhibits HIV-1 revealed that 5-azacytidine exerts antiviral activity through its incorporation into both viral RNA and DNA [95]. The antiviral activity of the derivative 5-aza-2′-deoxycytidine was detected using other retroviruses such as HTLV-1 [96]. Decitabine was successfully used in a trial to reduce HIV infectivity [97]. Moreover, azacytidine showed antiviral activity against Rift Valley fever virus (RVFV) [91], human metapneumovirus (HMPV) [98], A/H5N1 [99], IAV subtype H3N2 (A/H3N2), laboratory-adapted IAV H1N1 strains [100], EBV [101], and HSV-1 [102].

Zebularine (pyrimidin-2-one ribonucleoside), a cytidine analog that acts as a DNA demethylase inhibitor as well as a cytidine deaminase inhibitor, can be also considered as an antiviral drug [89]. However, the complex metabolism of zebularine and its limited DNA incorporation may restrain its applications in antiviral therapies. Indeed, zebularine is less potent than 5-azacytidine and its successful action requires higher doses for inhibition of DNMT activity [103].

Sinefungin (A9145) and its related metabolite, A9145C, are analogs of the methyl donor SAM and inhibitors of RNA and DNA methyltransferases [104]. Sinefungin (A9145) and its related metabolite, A9145C, were also found to be potent inhibitors of Newcastle disease virus (NDV), currently named Avian avulavirus 1 (AAvV-1) [105], Feline herpesvirus type 1 (FeHV-1) [106], vesicular stomatitis virus (VSV) [107], ZIKV [108], DENV [109], and the West Nile virus (WNV) [110]. It is worthwhile to mention that sinefungin has been identified as an inhibitor of 2′-O-RNA SARS-CoV-2 methyltransferase [111] and may electrostatically interact with the 2′-OH and N3 groups of adenosine moiety of RNA substrate thus mimicking the methyl transfer reaction of SAM substrate [112].

Tea polyphenol epigallocatechin-3-gallate (EGCG) is also another promising inhibitor of DNA/RNA methyltransferases. It was shown that EGCG can inhibit DNA methyltransferase and reactivate methylation-silenced genes in cancer cell lines [113].

Halby et al. developed a convergent synthetic pathway, starting from a protected bromomethylcytosine derivative, to synthesize transition state analogs of DNA methyltransferases [114]. However, they found low activity of seven 5-methylcytosine-adenosine compounds against hDNMT1, hDNMT3Acat, TRDMT1, and other human and viral RNA methyltransferases. Nevertheless, this research paves the way for the conception of new inhibitors based on the m^5^C scaffold.

## 7. Conclusions and Perspectives

In summary, we hypothesize that inhibitors of m^5^C RNA methyltransferases can be used in the future to modulate virus-mediated effects in host cells. However, several issues need to be addressed, namely, efficiency, selectivity, bioavailability, and biocompatibility of putative inhibitors in biological systems. The side effects of their use can be also a serious problem since these compounds can inhibit the enzyme activity of both virus-infected and virus-free cells. For instance, the use of 5-aza-2′-deoxycytidine can result in thrombocytopenia and anemia and less frequently can lead to congestive cardiac failure and tachycardia (http://sideeffects.embl.de). Moreover, it has been documented that high dose of azacytidine or 5-aza-2′-deoxycytidine may cause renal dysfunction [115] and spermatogenesis abnormalities [116] as well as may promote teratogenic effects during early embryogenesis [117]. Efficiency can be improved by molecular modeling of analogs in such way that the analogs are more effective than m^5^C in the binding to the catalytic sites of enzymes and the analogs are better matched structurally that may result in permanent inhibition and degradation of these enzymes [114]. Another problem may be the degradation of the analogs by extracellular and intracellular enzymes. Therefore, it is also important to develop systems that would prevent the degradation of the analogs before their targeted delivery. The selectivity of the inhibitors of m^5^C RNA methyltransferases can be improved by the addition of functional groups to the analogs [118]. A good solution would be to use the conjugates of m^5^C analogs based on the mechanism of cationic carriers that would allow for more efficient transport. Techniques for obtaining the oligonucleotide analogs with cationic backbone linkages were previously proposed [119]. Another well-established approach for nucleic acid delivery is based on cationic lipid-nucleic acid complexes [120,121]. The advantages of these systems for therapeutic applications include low toxicity and non-immunogenicity, ease of production, and the potential of transferring large amount of nucleic acids or oligonucleotides into the cells. Moreover, the addition of chemical groups to analogs can improve their bioavailability, e.g., during oral administration [122]. Improving the effectiveness of 5-azacytidine by increasing its bioavailability to human cells was also achieved by elaidic acid esterification [123]. An alternative delivery system for m^5^C analogs was also proposed, namely mesoporous silica nanoparticles [124]. In this view, the delivery of m^5^C RNA methyltransferase inhibitors can be improved by the use of nanocapsules that may serve as a promising technology for future antiviral therapies. The unique structure of nanocapsules may allow for proper loading of nucleoside analogs, oligonucleotides with nucleoside analogs, tag-like ligands, receptors, additional dyes, or other compounds that may support the monitoring and control of drug release. More studies are needed to better characterize the molecular bases of the biological action and therapeutic effects of m^5^C RNA methyltransferase inhibitors and their putative side effects in vitro and in vivo.

## Abbreviations

A/H5N1	influenza A virus subtype H5N1
A3G	apolipoprotein B mRNA editing enzyme catalytic subunit 3G (cytidine deaminase)
AAvV-1	avian avulavirus 1
ANG	angiogenin
APOBEC	apolipoprotein B editing complex
APOER2	apolipoprotein E receptor 2
CpG	5′C—phosphate—G3′
Cys	cysteine
DCV	*Drosophila* C virus
DENV	dengue virus
DNA	deoxyrybonucleic acid
DNMT2	DNA methyltransferase 2
dsRNA	double-stranded RNA
EBOV	Ebola virus
EBV	Epstein-Barr virus
EGCG	epigallocatechin-3-gallate
eRNA	enhancer RNA
FeHV-1	feline herpesvirus type 1
GEO	Gene Expression Omnibus
Glu	glutamic acid
Gly	glycine
HBV	hepatitis B virus
HCV	hepatitis C virus
hDNMT1	human DNA methyltransferase 1
hDNMT3Acat	human DNA methyltransferase 3A catalytic domain
HIV-1	human immunodeficiency virus 1
HMPV	human metapneumovirus
hnRNPK	heterogeneous nuclear ribonucleoprotein K
HPV	human papillomavirus
HSV-1	herpes simplex virus 1
HTLV	human T-lymphotropic virus
IAV	influenza A virus
IFN-β	interferon beta
KSHV	Kaposi’s sarcoma-associated herpesvirus
Leu	leucine
lncRNA	long non-coding RNA
m^5^C	5-methylcytosine
m^6^A	N6-methyladenosine
MDA-5	melanoma differentiation-associated protein 5
Met	methionine
miRNA	microRNA
MLV	murine leukemia virus
mRNA	messenger RNA
mt tRNA	mitochondrial tRNA
NDV	Newcastle disease virus
Nop2	nucleolar protein 2
Nsp3	non-structural protein 3
NSUN	NOL1/NOP2/SUN domain
nt	nucleotide
PGC-1α	peroxisome proliferator-activated receptor gamma coactivator 1-alpha
PKR	RNA-dependent protein kinase
P-TEFb	positive transcription elongation factor b
Rcm1	rRNA (cytosine-C5-)-methyltransferase
RNA	ribonucleic acid
rRNA	ribosomal RNA
RSV	respiratory syncytial virus
RVFV	Rift Valley fever virus
SAM	*S*-adenosylmethionine
SARS-Bat SRBD	SARS-CoV-like virus isolated from bats that contains the spike-protein receptor-binding domain from wild type SARS-CoV Urbani
SARS-CoV	severe acute respiratory syndrome coronavirus
SARS-CoV-2	severe acute respiratory syndrome coronavirus 2
snRNA	small nuclear RNA
+ssRNA	positive-sense single-stranded RNA
-ssRNA	negative-sense single-stranded RNA
ssRNA	single-stranded RNA
svRNA	small non-coding RNA fragments derived from vtRNA
svRNA	small viral RNA
TAR	transactivation response element
THOC4/ALYREF	THO complex subunit 4/Aly/REF export factor
Thr	threonine
tiRNA	tRNA-derived stress-induced RNA
TRDMT1	tRNA methyltransferase 1
TREX	transcription export complex
tRFs	tRNA-derived non-coding fragments
Trm4	multisite-specific tRNA:m^5^C-methyltransferase
tRNA	transfer RNA
UHPLC	ultrahigh-performance liquid chromatography
UPLC-MS/MS	ultra-performance liquid chromatography and tandem mass spectrometry
VSV	vesicular stomatitis virus
vtRNA	small vault RNA
WNV	West Nile virus
ZIKV	Zika virus

## References

[1] Boccaletto P., A Machnicka M., Purta E., Piątkowski P., Bagiński B., Wirecki T.K., De Crécy-Lagard V., Ross R., A Limbach P., Kotter A. (2018). MODOMICS: A database of RNA modification pathways. 2017 update. Nucleic Acids Res..

[2] Desrosiers R., Friderici K., Rottman F. (1974). Identification of Methylated Nucleosides in Messenger RNA from Novikoff Hepatoma Cells. Proc. Natl. Acad. Sci. USA.

[3] Amort T., Soulière M.F., Wille A., Jia X.-Y., Fiegl H., Wörle H., Micura R., Lusser A. (2013). Long non-coding RNAs as targets for cytosine methylation. RNA Biol..

[4] Amort T., Rieder D., Wille A., Khokhlova-Cubberley D., Riml C., Trixl L., Jia X.-Y., Micura R., Lusser A. (2017). Distinct 5-methylcytosine profiles in poly(A) RNA from mouse embryonic stem cells and brain. Genome Biol..

[5] Lewis C.J., Pan T., Kalsotra A. (2017). RNA modifications and structures cooperate to guide RNA–protein interactions. Nat. Rev. Mol. Cell Biol..

[6] Bohnsack M.T., Höbartner C., Bohnsack M.T. (2019). Eukaryotic 5-methylcytosine (m^5^C) RNA Methyltransferases: Mechanisms, Cellular Functions, and Links to Disease. Genes.

[7] Schaefer M., Pollex T., Hanna K., Lyko F. (2008). RNA cytosine methylation analysis by bisulfite sequencing. Nucleic Acids Res..

[8] Edelheit S., Schwartz S., Mumbach M.R., Wurtzel O., Sorek R. (2013). Transcriptome-Wide Mapping of 5-methylcytidine RNA Modifications in Bacteria, Archaea, and Yeast Reveals m^5^C within Archaeal mRNAs. PLoS Genet..

[9] Khoddami V., Cairns B.R. (2013). Identification of direct targets and modified bases of RNA cytosine methyltransferases. Nat. Biotechnol..

[10] Hussain S., Aleksic J., Blanco S., Dietmann S., Frye M. (2013). Characterizing 5-methylcytosine in the mammalian epitranscriptome. Genome Biol..

[11] Trixl L., Lusser A. (2018). The dynamic RNA modification 5-methylcytosine and its emerging role as an epitranscriptomic mark. Wiley Interdiscip. Rev. RNA.

[12] Dang W., Xie Y., Cao P., Xin S., Wang J., Li S., Li Y., Lu J. (2019). N6-Methyladenosine and Viral Infection. Front. Microbiol..

[13] Manners O., Baquero-Perez B., Whitehouse A. (2019). m^6^A: Widespread regulatory control in virus replication. Biochim. Biophys. Acta (BBA)-Bioenerg..

[14] Daffis S., Szretter K.J., Schriewer J., Li J., Youn S., Errett J., Lin T.-Y., Schneller S.W., Zust R., Dong H. (2010). 2′-O methylation of the viral mRNA cap evades host restriction by IFIT family members. Nat. Cell Biol..

[15] Pereira-Montecinos C., Valiente-Echeverría F., Soto-Rifo R. (2017). Epitranscriptomic regulation of viral replication. Biochim. Biophys. Acta (BBA)-Bioenerg..

[16] Reid R., Greene P.J., Santi D.V. (1999). Exposition of a family of RNA m^5^C methyltransferases from searching genomic and proteomic sequences. Nucleic Acids Res..

[17] Liu Y., Santi D.V. (2000). m^5^C RNA and m^5^C DNA methyl transferases use different cysteine residues as catalysts. Proc. Natl. Acad. Sci. USA.

[18] King M.Y., Redman K.L. (2002). RNA Methyltransferases Utilize Two Cysteine Residues in the Formation of 5-Methylcytosine. Biochemistry.

[19] Bourgeois G., Ney M., Gaspar I., Aigueperse C., Schaefer M., Kellner S., Helm M., Motorin Y. (2015). Eukaryotic rRNA Modification by Yeast 5-Methylcytosine-Methyltransferases and Human Proliferation-Associated Antigen p120. PLoS ONE.

[20] Gkatza N.A., Castro C., Harvey R.F., Heiß M., Popis M.C., Blanco S., Bornelöv S., Sajini A.A., Gleeson J.G., Griffin J.L. (2019). Cytosine-5 RNA methylation links protein synthesis to cell metabolism. PLoS Biol..

[21] Auxilien S., Guérineau V., Szweykowska-Kulinska Z., Golinelli-Pimpaneau B. (2012). The human tRNA m^5^C methyltransferase Misu is multisite-specific. RNA Biol..

[22] Van Haute L., Lee S.-Y., McCann B.J., A Powell C., Bansal D., Vasiliauskaitė L., Garone C., Shin S., Kim J.-S., Frye M. (2019). NSUN2 introduces 5-methylcytosines in mammalian mitochondrial tRNAs. Nucleic Acids Res..

[23] Brzezicha B., Schmidt M., Makałowska I., Jarmołowski A., Pieńkowska J., Szweykowska-Kulińska Z. (2006). Identification of human tRNA:m^5^C methyltransferase catalysing intron-dependent m^5^C formation in the first position of the anticodon of the. Nucleic Acids Res..

[24] Chen X., Li A., Sun B.-F., Yang Y., Han Y.-N., Yuan X., Chen R.-X., Wei W.-S., Liu Y., Gao C.-C. (2019). 5-methylcytosine promotes pathogenesis of bladder cancer through stabilizing mRNAs. Nat. Cell Biol..

[25] Haag S., Sloan K.E., Ranjan N., Warda A.S., Kretschmer J., Blessing C., Hubner B., Seikowski J., Dennerlein S., Rehling P. (2016). NSUN3 and ABH1 modify the wobble position of mt-tRNAMet to expand codon recognition in mitochondrial translation. EMBO J..

[26] Nakano S., Suzuki T., Kawarada L., Iwata H., Asano K. (2016). NSUN3 methylase initiates 5-formylcytidine biogenesis in human mitochondrial tRNA(Met). Nat. Chem. Biol..

[27] Metodiev M.D., Spåhr H., Polosa P.L., Meharg C., Becker C., Altmueller J., Habermann B., Larsson N.-G., Ruzzenente B. (2014). NSUN4 Is a Dual Function Mitochondrial Protein Required for Both Methylation of 12S rRNA and Coordination of Mitoribosomal Assembly. PLoS Genet..

[28] Sharma S., Yang J., Watzinger P., Kötter P., Entian K.-D. (2013). Yeast Nop2 and Rcm1 methylate C2870 and C2278 of the 25S rRNA, respectively. Nucleic Acids Res..

[29] Janin M., Ortiz-Barahona V., De Moura M.C., Martínez-Cardús A., Llinàs-Arias P., Soler M., Nachmani D., Pelletier J., Schumann U., Calleja-Cervantes M.E. (2019). Epigenetic loss of RNA-methyltransferase NSUN5 in glioma targets ribosomes to drive a stress adaptive translational program. Acta Neuropathol..

[30] Heissenberger C., Liendl L., Nagelreiter F., Gonskikh Y., Yang G., Stelzer E.M., Krammer T.L., Micutkova L., Vogt S., Kreil D.P. (2019). Loss of the ribosomal RNA methyltransferase NSUN5 impairs global protein synthesis and normal growth. Nucleic Acids Res..

[31] Haag S., Warda A.S., Kretschmer J., Günnigmann M.A., Höbartner C., Bohnsack M.T. (2015). NSUN6 is a human RNA methyltransferase that catalyzes formation of m^5^C72 in specific tRNAs. RNA.

[32] Liu R.-J., Long T., Li J., Li H., Wang E.-D. (2017). Structural basis for substrate binding and catalytic mechanism of a human RNA:m^5^C methyltransferase NSun6. Nucleic Acids Res..

[33] Long T., Li J., Li H., Zhou M., Zhou X.-L., Liu R.-J., Wang E.-D. (2016). Sequence-specific and Shape-selective RNA Recognition by the Human RNA 5-Methylcytosine Methyltransferase NSun6. J. Biol. Chem..

[34] Aguilo F., Li S., Balasubramaniyan N., Sancho A., Benko S., Zhang F., A Vashisht A., Rengasamy M., Andino B., Chen C.-H. (2016). Deposition of 5-Methylcytosine on Enhancer RNAs Enables the Coactivator Function of PGC-1α. Cell Rep..

[35] Goll M.G., Kirpekar F., Maggert K.A., Yoder J.A., Hsieh C.-L., Zhang X., Golic K.G., Jacobsen S.E., Bestor T.H. (2006). Methylation of tRNAAsp by the DNA Methyltransferase Homolog Dnmt2. Science.

[36] Schaefer M., Lyko F. (2010). Lack of evidence for DNA methylation of Invader4 retroelements in *Drosophila* and implications for Dnmt2-mediated epigenetic regulation. Nat. Genet..

[37] Cui W., Pizzollo J., Han Z., Marcho C., Zhang K., Mager J. (2015). Nop2 is required for mammalian preimplantation development. Mol. Reprod. Dev..

[38] Hussain S., Tuorto F., Menon S., Blanco S., Cox C., Flores J.V., Watt S., Kudo N.R., Lyko F., Frye M. (2013). The Mouse Cytosine-5 RNA Methyltransferase NSun2 Is a Component of the Chromatoid Body and Required for Testis Differentiation. Mol. Cell. Biol..

[39] Tuorto F., Liebers R., Musch T., Schaefer M., Hofmann S., Kellner S., Frye M., Helm M., Stoecklin G., Lyko F. (2012). RNA cytosine methylation by Dnmt2 and NSun2 promotes tRNA stability and protein synthesis. Nat. Struct. Mol. Biol..

[40] Zhang T., Chen P., Li W., Sha S., Wang Y., Yuan Z., Shen B., Chen L. (2019). Cognitive deficits in mice lacking Nsun5, a cytosine-5 RNA methyltransferase, with impairment of oligodendrocyte precursor cells. Glia.

[41] Selmi T., Hussain S., Dietmann S., Heiss M., Carter J.-M., Dennison R., Flad S., Huang Y.-L., Kellner S., Borneloev S. (2020). Sequence- and structure-specific cytosine-5 mRNA methylation by NSUN6. bioRxiv.

[42] Harris T., Marquez B., Suarez S., Schimenti J. (2007). Sperm Motility Defects and Infertility in Male Mice with a Mutation in Nsun7, a Member of the Sun Domain-Containing Family of Putative RNA Methyltransferases1. Biol. Reprod..

[43] Dubin D.T., Stollar V. (1975). Methylation of Sindbis virus “26S” messenger RNA. Biochem. Biophys. Res. Commun..

[44] Dubin D.T., Stollar V., HsuChen C.-C., Timko K., Guild G.M. (1977). Sindbis virus messenger RNA: The 5′-termini and methylated residues of 26 and 42 S RNA. Virology.

[45] Sommer S., Salditt-Georgieff M., Bachenheimer S., Darnell J., Furuichi Y., Morgan M., Shatkin A. (1976). The methylation of adenovirus-specific nuclear and cytoplasmic RNA. Nucleic Acids Res..

[46] Courtney D.G., Chalem A., Bogerd H.P., Law B.A., Kennedy E.M., Holley C.L., Cullen B.R. (2019). Extensive Epitranscriptomic Methylation of A and C Residues on Murine Leukemia Virus Transcripts Enhances Viral Gene Expression. mBio.

[47] Courtney D., Tsai K., Bogerd H.P., Kennedy E.M., Law B.A., Emery A., Swanstrom R., Holley C.L., Cullen B.R. (2019). Epitranscriptomic Addition of m^5^C to HIV-1 Transcripts Regulates Viral Gene Expression. SSRN Electron. J..

[48] Rima B.K., McFerran N.V. (1997). Dinucleotide and stop codon frequencies in single-stranded RNA viruses. J. Gen. Virol..

[49] Karlin S., Doerfler W., Cardon L.R. (1994). Why is CpG suppressed in the genomes of virtually all small eukaryotic viruses but not in those of large eukaryotic viruses?. J. Virol..

[50] Hoelzer K., Shackelton L.A., Parrish C.R. (2008). Presence and role of cytosine methylation in DNA viruses of animals. Nucleic Acids Res..

[51] Cheng X., Virk N., Chen W., Ji S., Ji S., Sun Y., Wu X. (2013). CpG Usage in RNA Viruses: Data and Hypotheses. PLoS ONE.

[52] Matyasek R., Kovarik A. (2020). Mutation patterns of human SARS-COV-2 and Bat RATG13 coronavirus genomes are strongly biased towards C>U transitions, indicating rapid evolution in their hosts. Genes.

[53] Viehweger A., Krautwurst S., Lamkiewicz K., Madhugiri R., Ziebuhr J., Hölzer M., Marz M. (2019). Direct RNA nanopore sequencing of full-length coronavirus genomes provides novel insights into structural variants and enables modification analysis. Genome Res..

[54] Chiang C.-M., D K., Jy L., Js Y., Jw K., Vn K., H C. (2020). Faculty Opinions recommendation of The Architecture of SARS-CoV-2 Transcriptome. Fac. Opin. Post Publ. Peer Rev. Biomed. Lit..

[55] Taiaroa G., Rawlinson D., Featherstone L., Pitt M., Caly L., Druce J., Purcell D., Harty L., Tran T., Roberts J. (2020). Direct RNA sequencing and early evolution of SARS-CoV-2 2020. bioRxiv.

[56] Sawicki S.G., Sawicki D.L. (1995). Coronaviruses use Discontinuous Extension for Synthesis of Subgenome-Length Negative Strands. Adv. Exp. Med. Biol..

[57] Zúñiga S., Sola I., Alonso S., Enjuanes L. (2004). Sequence Motifs Involved in the Regulation of Discontinuous Coronavirus Subgenomic RNA Synthesis. J. Virol..

[58] Sawicki S.G., Sawicki D.L., Siddell S.G. (2006). A Contemporary View of Coronavirus Transcription. J. Virol..

[59] Antzin-Anduetza I., Mahiet C., Granger L.A., Odendall C., Swanson C.M. (2017). Increasing the CpG dinucleotide abundance in the HIV-1 genomic RNA inhibits viral replication. Retrovirology.

[60] Wasson M.K., Borkakoti J., Kumar A., Biswas B., Vivekanandan P. (2017). The CpG dinucleotide content of the HIV-1 envelope gene may predict disease progression. Sci. Rep..

[61] Trus I., Udenze D., Berube N., Wheler C., Martel M.-J., Gerdts V., Karniychuk U. (2020). CpG-Recoding in Zika Virus Genome Causes Host-Age-Dependent Attenuation of Infection With Protection Against Lethal Heterologous Challenge in Mice. Front. Immunol..

[62] Durdevic Z., Schaefer M. (2013). Dnmt2 methyltransferases and immunity: An ancient overlooked connection between nucleotide modification and host defense?. BioEssays.

[63] Durdevic Z., Mobin M.B., Hanna K., Lyko F., Schaefer M. (2013). The RNA Methyltransferase Dnmt2 Is Required for Efficient Dicer-2-Dependent siRNA Pathway Activity in *Drosophila*. Cell Rep..

[64] Lee J.S., Tabata K., Twu W.-I., Rahman S., Kim H.S., Yu J.B., Jee M.H., Bartenschlager R., Jang S.K. (2019). RACK1 mediates rewiring of intracellular networks induced by hepatitis C virus infection. PLoS Pathog..

[65] Stavrou S., Ross S.R. (2015). APOBEC3 Proteins in Viral Immunity. J. Immunol..

[66] Bishop K.N., Holmes R.K., Sheehy A.M., Davidson N.O., Cho S.-J., Malim M.H. (2004). Cytidine Deamination of Retroviral DNA by Diverse APOBEC Proteins. Curr. Biol..

[67] Beale R.C., Petersen-Mahrt S.K., Watt I.N., Harris R.S., Rada C., Neuberger M.S. (2004). Comparison of the Differential Context-dependence of DNA Deamination by APOBEC Enzymes: Correlation with Mutation Spectra in vivo. J. Mol. Biol..

[68] Kim E.-Y., Lorenzo-Redondo R., Little S.J., Chung Y.-S., Phalora P.K., Berry I.M., Archer J., Penugonda S., Fischer W., Richman D.D. (2014). Human APOBEC3 Induced Mutation of Human Immunodeficiency Virus Type-1 Contributes to Adaptation and Evolution in Natural Infection. PLoS Pathog..

[69] Wijesinghe P., Bhagwat A.S. (2012). Efficient deamination of 5-methylcytosines in DNA by human APOBEC3A, but not by AID or APOBEC3G. Nucleic Acids Res..

[70] McIntyre W., Netzband R., Bonenfant G., Biegel J.M., Miller C., Fuchs G., Henderson E., Arra M., Canki M., Fabris D. (2018). Positive-sense RNA viruses reveal the complexity and dynamics of the cellular and viral epitranscriptomes during infection. Nucleic Acids Res..

[71] Schwarz K.B. (1996). Oxidative stress during viral infection: A review. Free Radic. Biol. Med..

[72] Sloan K.E., Bohnsack M.T., Watkins N.J. (2013). The 5S RNP Couples p53 Homeostasis to Ribosome Biogenesis and Nucleolar Stress. Cell Rep..

[73] Kong W., Biswas A., Zhou D., Fiches G., Fujinaga K., Santoso N., Zhu J. (2020). Nucleolar protein Nop2/Nsun1 suppresses HIV-1 transcription and promotes viral latency by competing with TAT for TAR binding and methylation. PLoS Pathog..

[74] Shlomai A., Rechtman M.M., Burdelova E.O., Zilberberg A., Hoffman S., Solar I., Fishman S., Halpern Z., Sklan E.H. (2012). The metabolic regulator PGC-1α links hepatitis C virus infection to hepatic insulin resistance. J. Hepatol..

[75] Thiagarajan D., Dev R.R., Khosla S. (2011). The DNA methyltranferase Dnmt2 participates in RNA processing during cellular stress. Epigenetics.

[76] Dev R.R., Ganji R., Singh S.P., Mahalingam S., Banerjee S., Khosla S. (2017). Cytosine methylation by DNMT2 facilitates stability and survival of HIV-1 RNA in the host cell during infection. Biochem. J..

[77] Mitchell H.D., Eisfeld A.J., Sims A.C., McDermott J.E., Matzke M.M., Webb-Robertson B.J., Tilton S.C., Tchitchek N., Josset L., Li C. (2013). A network integration approach to predict conserved regulators related to pathogenicity of influenza and SARS-COV respiratory viruses. PLoS ONE.

[78] Linder A., Hornung V. (2018). Mitochondrial dsRNA: A New DAMP for MDA5. Dev. Cell.

[79] Dhir A., Dhir S., Borowski L.S., Jimenez L., Teitell M., Rötig A., Crow Y.J., Rice G.I., Duffy D., Tamby C. (2018). Mitochondrial double-stranded RNA triggers antiviral signalling in humans. Nat. Cell Biol..

[80] Cheng J.X., Chen L., Li Y., Cloe A., Yue M., Wei J., Watanabe K.A., Shammo J.M., Anastasi J., Shen Q.J. (2018). RNA cytosine methylation and methyltransferases mediate chromatin organization and 5-azacytidine response and resistance in leukaemia. Nat. Commun..

[81] Ivanov P., Emara M.M., Villen J., Gygi S.P., Anderson P. (2011). Angiogenin-Induced tRNA Fragments Inhibit Translation Initiation. Mol. Cell.

[82] Goodarzi H., Liu X., Nguyen H.C., Zhang S., Fish L., Tavazoie S.F. (2015). Endogenous tRNA-Derived Fragments Suppress Breast Cancer Progression via YBX1 Displacement. Cell.

[83] Gebetsberger J.V., Wyss L., Mleczko A.M., Reuther J., Polacek N. (2017). A tRNA-derived fragment competes with mRNA for ribosome binding and regulates translation during stress. RNA Biol..

[84] Deng J., Ptashkin R.N., Chen Y., Cheng Z., Liu G., Phan T., Deng X., Zhou J., Lee I., Lee Y.S. (2015). Respiratory Syncytial Virus Utilizes a tRNA Fragment to Suppress Antiviral Responses Through a Novel Targeting Mechanism. Mol. Ther..

[85] Yang X., Yang Y., Sun B.-F., Chen Y.-S., Xu J.-W., Lai W.-Y., Li A., Wang X., Bhattarai D.P., Xiao W. (2017). 5-methylcytosine promotes mRNA export — NSUN2 as the methyltransferase and ALYREF as an m^5^C reader. Cell Res..

[86] Boyne J.R., Colgan K.J., Whitehouse A. (2008). Recruitment of the complete HTREX complex is required for Kaposi’s sarcoma-associated herpesvirus intronless mRNA nuclear export and virus replication. PLoS Pathog..

[87] Hussain S., Sajini A.A., Blanco S., Dietmann S., Lombard P., Sugimoto Y., Paramor M., Gleeson J.G., Odom D.T., Ule J. (2013). NSun2-Mediated Cytosine-5 Methylation of Vault Noncoding RNA Determines Its Processing into Regulatory Small RNAs. Cell Rep..

[88] Li F., Chen Y., Zhang Z., Ouyang J., Wang Y., Yan R., Huang S., Gao G.F., Guo G., Chen J.-L. (2015). Robust expression of vault RNAs induced by influenza a virus plays a critical role in suppression of PKR-mediated innate immunity. Nucleic Acids Res..

[89] McAllister C.S., Taghavi N., Samuel C.E. (2012). Protein Kinase PKR Amplification of Interferon β Induction Occurs through Initiation Factor eIF-2α-mediated Translational Control. J. Biol. Chem..

[90] Morales L., Oliveros J.C., Fernandez-Delgado R., Tenoever B.R., Enjuanes L., Sola I. (2017). SARS-CoV-Encoded Small RNAs Contribute to Infection-Associated Lung Pathology. Cell Host Microbe.

[91] Ianevski A., Zusinaite E., Kuivanen S., Strand M., Lysvand H., Teppor M., Kakkola L., Paavilainen H., Laajala M., Kallio-Kokko H. (2018). Novel activities of safe-in-human broad-spectrum antiviral agents. Antivir. Res..

[92] Šorm F., Pískala A., Čihák A., Veselý J. (1964). 5-Azacytidine, a new, highly effective cancerostatic. Cell. Mol. Life Sci..

[93] Schaefer M., Hagemann S., Hanna K., Lyko F. (2009). Azacytidine Inhibits RNA Methylation at DNMT2 Target Sites in Human Cancer Cell Lines. Cancer Res..

[94] Bouchard J., Walker M.C., Leclerc J.M., Lapointe N., Beaulieu R., Thibodeau L. (1990). 5-azacytidine and 5-azadeoxycytidine inhibit human immunodeficiency virus type 1 replication in vitro. Antimicrob. Agents Chemother..

[95] Dapp M.J., Clouser C.L., Patterson S., Mansky L.M. (2009). 5-Azacytidine Can Induce Lethal Mutagenesis in Human Immunodeficiency Virus Type 1. J. Virol..

[96] Diamantopoulos P.T., Michael M., Benopoulou O., Bazanis E., Tzeletas G., Meletis J., Vayopoulos G., Viniou N.-A. (2012). Antiretroviral activity of 5-azacytidine during treatment of a HTLV-1 positive myelodysplastic syndrome with autoimmune manifestations. Virol. J..

[97] Clouser C.L., Patterson S.E., Mansky L.M. (2010). Exploiting Drug Repositioning for Discovery of a Novel HIV Combination Therapy. J. Virol..

[98] Bösl K., Ianevski A., Than T.T., Andersen P.I., Kuivanen S., Teppor M., Zusinaite E., Dumpis U., Vitkauskiene A., Cox R.J. (2019). Common Nodes of Virus–Host Interaction Revealed Through an Integrated Network Analysis. Front. Immunol..

[99] Huang F., Zhang C., Liu Q., Zhao Y., Zhang Y., Qin Y., Li X., Li C., Zhou C., Jin N. (2020). Identification of amitriptyline HCl, flavin adenine dinucleotide, azacitidine and calcitriol as repurposing drugs for influenza A H5N1 virus-induced lung injury. PLoS Pathog..

[100] Pauly M.D., Lauring A.S. (2015). Effective Lethal Mutagenesis of Influenza Virus by Three Nucleoside Analogs. J. Virol..

[101] Chan A.T.C., Tao Q., Robertson K.D., Flinn I.W., Mann R.B., Klencke B., Kwan W.H., Leung T.W.-T., Johnson P.J., Ambinder R.F. (2004). Azacitidine Induces Demethylation of the Epstein-Barr Virus Genome in Tumors. J. Clin. Oncol..

[102] Takimoto K. (1985). Reactivation and mutagenesis of herpes virus in 5-azacytidine-treated monkey kidney cells. Mutat. Res. Repair Rep..

[103] Rao S.P., Rechsteiner M.P., Berger C., Sigrist J.A., Nadal D., Bernasconi M. (2007). Zebularine reactivates silenced *E-cadherin* but unlike 5-azacytidine does not induce switching from latent to lytic Epstein-Barr virus infection in Burkitt’s lymphoma Akata cells. Mol. Cancer.

[104] Yebra M.J., Sánchez J., Martin C.G., Hardisson C., Barbes C. (1991). The effect of sinefungin and synthetic analogues on RNA and DNA methyltransferases from *Streptomyces*. J. Antibiot..

[105] Pugh C.S., Borchardt R.T., O Stone H. (1978). Sinefungin, a potent inhibitor of virion mRNA(guanine-7-)-methyltransferase, mRNA(nucleoside-2′-)-methyltransferase, and viral multiplication. J. Biol. Chem..

[106] Kuroda Y., Yamagata H., Nemoto M., Inagaki K., Tamura T., Maeda K. (2019). Antiviral effect of sinefungin on in vitro growth of feline herpesvirus type 1. J. Antibiot..

[107] Li J., Chorba J.S., Whelan S.P. (2007). Vesicular Stomatitis Viruses Resistant to the Methylase Inhibitor Sinefungin Upregulate RNA Synthesis and Reveal Mutations That Affect mRNA Cap Methylation. J. Virol..

[108] Hercik K., Brynda J., Nencka R., Boura E. (2017). Structural basis of Zika virus methyltransferase inhibition by sinefungin. Arch. Virol..

[109] Lim S.P., Sonntag L.S., Noble C., Nilar S.H., Ng R.H., Zou G., Monaghan P., Chung K.Y., Dong H., Liu B. (2010). Small Molecule Inhibitors That Selectively Block Dengue Virus Methyltransferase. J. Biol. Chem..

[110] Chen H., Liu L., Jones S.A., Banavali N., Kass J., Li Z., Zhang J., Kramer L.D., Ghosh A.K., Li H. (2013). Selective inhibition of the West Nile virus methyltransferase by nucleoside analogs. Antivir. Res..

[111] Krafcikova P., Silhan J., Nencka R., Boura E. (2020). Structural analysis of the SARS-COV-2 methyltransferase complex involved in RNA cap creation bound to sinefungin. Nat. Commun..

[112] Mahalapbutr P., Kongtaworn N., Rungrotmongkol T. (2020). Structural insight into the recognition of S-adenosyl-L-homocysteine and sinefungin in SARS-COV-2 NSP16/NSP10 RNA cap 2′-O-methyltransferase. Comput. Struct. Biotechnol. J..

[113] Fang M.Z., Wang Y., Ai N., Hou Z., Sun Y., Lu H., Welsh W., Yang C.S. (2003). Tea polyphenol (-)-epigallocatechin-3-gallate inhibits DNA methyltransferase and reactivates methylation-silenced genes in cancer cell lines. Cancer Res..

[114] Halby L., Marechal N., Pechalrieu D., Cura V., Franchini D.-M., Faux C., Alby F., Troffer-Charlier N., Kudithipudi S., Jeltsch A. (2018). Hijacking DNA methyltransferase transition state analogues to produce chemical scaffolds for PRMT inhibitors. Philos. Trans. R. Soc. B Biol. Sci..

[115] Kintzel P.E. (2001). Anticancer Drug—Induced Kidney Disorders. Drug Saf..

[116] Oakes C.C., Kelly T.L.J., Robaire B., Trasler J.M. (2007). Adverse Effects of 5-Aza-2′-Deoxycytidine on Spermatogenesis Include Reduced Sperm Function and Selective Inhibition of *de Novo* DNA Methylation. J. Pharmacol. Exp. Ther..

[117] Branch S., Francis B.M., Brownie C.F., Chernoff N. (1996). Teratogenic effects of the demethylating agent 5-aza-2′-deoxycytidine in the Swiss Webster mouse. Toxicology.

[118] Valderrama J.A., González M.F., Colonelli P., Vásquez-Velásquez D. (2006). Design and Synthesis of Angucyclinone 5-Aza Analogues. Synlett.

[119] Meng M., Ducho C. (2018). Oligonucleotide analogues with cationic backbone linkages. Beilstein J. Org. Chem..

[120] Wang J., Ayano E., Maitani Y., Kanazawa H. (2017). Enhanced cellular uptake and gene silencing activity of siRNA using temperature-responsive polymer-modified liposome. Int. J. Pharm..

[121] Safinya C.R., Ewert K.K., Majzoub R.N., Leal C. (2014). Cationic liposome–nucleic acid complexes for gene delivery and gene silencing. New J. Chem..

[122] Ziemba A., Hayes E., Freeman B.B., Ye T., Pizzorno G. (2011). Development of an Oral Form of Azacytidine: 2′3′5′Triacetyl-5-Azacytidine. Chemother. Res. Pract..

[123] Brueckner B., Rius M., Markelova M.R., Fichtner I., Hals P.-A., Sandvold M.L., Lyko F. (2010). Delivery of 5-Azacytidine to Human Cancer Cells by Elaidic Acid Esterification Increases Therapeutic Drug Efficacy. Mol. Cancer Ther..

[124] Cheng J., Ding Q., Wang J., Deng L., Yang L., Tao L., Lei H., Lu S. (2016). 5-Azacytidine delivered by mesoporous silica nanoparticles regulates the differentiation of P19 cells into cardiomyocytes. Nanoscale.

